# Expectation, Attitude, and Barriers to Receiving Telehomecare Among Caregivers of Homebound or Bedridden Older Adults: Qualitative Study

**DOI:** 10.2196/48132

**Published:** 2024-02-07

**Authors:** Pansiree Onseng, Wichuda Jiraporncharoen, Sasiwimon Moonkayaow, Pimchai Veerasirikul, Nutchar Wiwatkunupakarn, Chaisiri Angkurawaranon, Kanokporn Pinyopornpanish

**Affiliations:** 1 Department of Family Medicine Chiang Mai University Chiang Mai Thailand; 2 Global Health and Chronic Conditions Research Group Chiang Mai University Chiang Mai Thailand

**Keywords:** telehomecare, telemedicine, telehealth, caregivers, older adults, attitudes

## Abstract

**Background:**

In recent years, telehomecare has become an increasingly important option for health care providers to deliver continuous care to their patients.

**Objective:**

This study aims to explore the expectations, attitudes, and barriers to telehomecare among caregivers of homebound or bedridden older adults.

**Methods:**

This qualitative study used semistructured interviews to explore caregivers’ perspectives on telehomecare for homebound or bedridden older adults. The study adhered to the SRQR (Standards for Reporting Qualitative Research) guidelines. Participants were selected using convenience sampling from caregivers of homebound or bedridden older adults with experience in both in-person home visits and telehomecare services provided by the Department of Family Medicine at Chiang Mai University, in an urban area of Chiang Mai Province in Northern Thailand. Semistructured interviews were conducted. The interviews were audio recorded with participant consent and transcribed verbatim. The framework method was used, involving multiple readings of transcripts to facilitate familiarization and accuracy checking. The study used the technology acceptance model and comprehensive geriatric assessment as the analytical framework.

**Results:**

The study included 20 caregivers of older adult patients. The patients were predominantly female (15/20, 75%), with an average age of 86.2 years. Of these patients, 40% (n=8) of patients were bedridden, and 60% (n=12) of patients were homebound. Caregivers expressed generally positive attitudes toward telehomecare. They considered it valuable for overall health assessment, despite recognizing certain limitations, particularly in physical assessments. Psychological assessments were perceived as equally effective. While in-person visits offered more extensive environmental assessments, caregivers found ways to make telehomecare effective. Telehomecare facilitated multidisciplinary care, enabling communication with specialists. Caregivers play a key role in care planning and adherence. Challenges included communication issues due to low volume, patient inattention, and faulty devices and internet signals. Some caregivers helped overcome these barriers. The loss of information was mitigated by modifying signaling equipment. Technology use was a challenge for some older adult caregivers. Despite these challenges, telehomecare offered advantages in remote communication and resolving scheduling conflicts. Caregivers varied in their preferences. Some preferred in-person visits for a broader view, while others favored telehomecare for its convenience. Some had no strong preference, appreciating both methods, while others considered the situation and patient conditions when choosing between them. Increased experience with telehomecare led to more confidence in its use.

**Conclusions:**

Caregivers have positive attitudes and high expectations for telehomecare services. Although there may be barriers to receiving care through this mode, caregivers have demonstrated the ability to overcome these challenges, which has strengthened their confidence in telehomecare. However, it is important to enhance the skills of caregivers and health care teams to overcome barriers and optimize the use of telehomecare.

## Introduction

Thai society has come to recognize the challenge of the aging society and that there is a rapid growth in the number of homebound or bedridden older adult patients with chronic diseases [1]. Homebound refers to a state in which one’s life space is limited to their residence, yet they retain the ability to move within their home. On the other hand, being bedridden is a condition in which a person has experienced significant physical weakness and can no longer move about freely, thus being confined to their bed. These 2 statuses are typically caused by the patient’s comorbidities and often result in the need for comprehensive care and caregiver [2-4]. As a result, coordinated health care services are essential for these individuals, and home health care services have been found to be effective in improving patient outcomes. A long-term care policy has been implemented to provide home care and social support for this population [1,5]. These services are particularly useful for doctors to assess a patient’s living conditions and have been shown to decrease hospitalization, improve physical and psychosocial health, and enhance the overall quality of life for older adults [6,7].

Telemedicine has emerged as a potential solution to bridge the gap between homebound or bedridden patients and health care services [8]. By allowing health care providers to connect with patients remotely, telemedicine can reduce travel costs, provide convenience, and help control the spread of the pandemic [9]. However, there are also disadvantages and challenges associated with this technology, such as the lack of face-to-face communication between doctors and patients and the need for patients to have the technical skills to use these devices [1]. These factors may make the home visit system unsustainable [10].

Over the past 3 years, the COVID-19 pandemic has further highlighted the need for telemedicine, as in-person home visits have been interrupted and transformed into telehomecare for patients requiring continuous health care at home [11-13]. Telehomecare is a form of telemedicine that combines videoconferencing and health monitoring between homebound or bedridden patients and home health care providers [14]. In order to conduct telehomecare for homebound or bedridden older adult care, the caregiver has the main role of helping monitor the patient and to communicate all information to health care team. Therefore, if the caregivers are also older adults and are not proficient with technology, this can pose a barrier, especially for patients living in remote areas [15].

Prior studies on the perception or perspective of the use of telemedicine in caring for older adults have primarily focused on the viewpoints of physicians [16,17] and older adult patients [18,19], rather than caregivers. Physicians have also reported perceiving advantages in the use of telemedicine for older adults, including the reduction of postponed care, the promotion of timely care, enhanced physician efficiency, improved communication with both patients and caregivers, the alleviation of patient travel burdens, and the facilitation of health outreach and educational efforts [17]. The use of technology for health care in older adults could be influenced by their age and the complexity of diseases they face [19], impacting their acceptance of technology [20] and use behavior [21]. It has been noted that the technological acceptance of patients and providers appears to have positive effects on patient outcomes, including self-management and readmissions. The literature also indicates that family caregivers play a vital role in assisting the patient’s decision to adopt and engage with technology [22]. Physicians are aware that caregivers have a role in deciding whether to use telehealth services [16]. Overall, it is important to assess caregivers’ genuine perspectives and suggestions.

A recent study from the United States has explored family caregivers’ perceived benefits and barriers to telemedicine visits for community-dwelling older adults with chronic diseases [23]. Caregivers reported effectiveness in increasing access and continuity of care, but they also expressed concerns about their older relatives’ ability to access and use the technology independently and difficulty in establishing patient-provider rapport. However, these results could be different in Thailand and among families of older adults with more limited capacity, such as the homebound or bedridden population. Thus, this study aims to investigate the expectations, attitudes, and barriers concerning telehomecare among caregivers of homebound or bedridden older adults. Specifically, it will explore caregivers’ expectations regarding the perceived usefulness of the service in delivering comprehensive care for older adults with limited mobility; the barriers that affect the perceived ease of using the service; and their overall attitudes toward the care provided, including their preferences. The results of this study will provide useful insights for the creation of effective care plans for homebound or bedridden older adult patients and their caregivers.

## Methods

### Study Design

This study was a qualitative study, using semistructured interviews to investigate the perspectives of using telehomecare for homebound or bedridden older adults, as perceived by their caregivers. The study was reported according to the SRQR (Standards for Reporting Qualitative Research) guidelines [24].

### Ethical Considerations

Ethical approval was obtained through the Medical Ethical Committee of Chiang Mai University, Chiang Mai, Thailand (227/2021). Informed consent was obtained from all participants. The privacy and confidentiality of human participants were rigorously protected throughout this study. Data were either anonymized or deidentified, as applicable, to safeguard the identity of participants. Participants in the study received a monetary compensation of ฿100 (US $2.7) for their participation.

### Recruitment

Participants were selected using convenience sampling from caregivers of homebound or bedridden older adult patients who had experience with both in-person home visits and telehomecare services provided by the Department of Family Medicine at Chiang Mai University. The samples were selected from a list of families of older adults who had received care within the past 2 months. Research assistants reached out to these families via phone calls until the total expected sample size was achieved. The study was conducted in an urban area of Chiang Mai Province in Northern Thailand. Inclusion criteria were that participants must be the primary caregiver and speak Thai, while caregivers with communication difficulties were excluded. The researchers expected to recruit at least 20 participants to achieve data saturation, based on similar qualitative studies [25].

### Data Collection

Semistructured in-depth interviews were conducted between November 2021 and March 2022. There were two parts to data collection as follows: (1) participant characteristics and (2) in-depth interviews about the perspectives of using telehomecare for homebound or bedridden older adults. The patient interviews were conducted in Thai by a single researcher (PO) who was trained in the interview method and interview questions by the qualitative researchers (KP and WJ). Each interview was conducted in a private counseling room to help ensure comfort and confidentiality. Each interview was audio recorded with the participant’s consent, followed by verbatim transcription. PO and KP subsequently reviewed the audio record, and any issues that needed further exploration were discussed. Participant characteristics including sex, age, occupation, relationship with the patient, and the duration of care as the caregiver were recorded. Patient diagnoses and homebound or bedridden status were also gathered. Open-ended interview guides were designed and then piloted to make sure that participants were allowed to share their thoughts freely. The interview guide was described in Textbox 1.

**The interview guide.**

- How long has your family been receiving home care and telehomecare?
- What was the main reason for switching from in-person care to telehomecare?
- Do you perceive telehomecare as useful for your family?
- As a caregiver for an older adult patient, do you feel confident about receiving care for the patient through telehomecare?
- Can you compare the effectiveness of care between in-person home visits and telehomecare for older adult patients?
- Are you satisfied with these services? Which one do you prefer and why?
- What do you see as potential barriers or concerns regarding the use of telehomecare for caring for older adult patients?

### Data Analysis

We followed the framework method for the analysis of qualitative data [26]. Each transcript was read multiple times by the 2 researchers (PO and KP) to aid familiarization and to check the accuracy of each transcript. The key elements of the technology acceptance model and comprehensive geriatric assessment for older adult patient care were used as the analytical framework to help identify key issues, concepts, and themes. The study aimed to address the 2 main research questions, which align with the technology acceptance model in the context of providing care for older adults from the perspective of caregivers. The technology acceptance model is the theory that suggests the predictors toward the acceptance and rejection to use technology. These predictors include (1) perceived usefulness and (2) perceived ease of use which are influenced by, for example, technology anxiety or experience, subjective norms, expectations, trust, cultural diversities, and technology characteristics [27]. These factors, in turn, shape attitudes toward using telehomecare services.

The first question focused on perceived usefulness (expectation), incorporating comprehensive geriatric assessment to determine whether telehomecare is suitable for providing comprehensive home-based care for older adults. Comprehensive geriatric assessment is usually carried out while providing care for older adults at home to increase the understanding of an older person’s care needs and preferences and to help in finding common goals of care [28,29]. Its three key elements include (1) comprehensiveness (physical, psychological, and environmental issues); (2) multidisciplinarity; and (3) person‐centered goal setting. The second question concentrated on the perceived ease of use of telehomecare for caregivers caring for older adults. This encompassed concerns and perceived barriers.

To explore our research questions, we adopted the framework method, which used a combined approach, incorporating both deductive and inductive elements for the analysis [26]. The deductive approach was applied to the preexisting theories, specifically the technology acceptance model and comprehensive geriatric assessment. Meanwhile, the inductive approach was used to analyze the expectations, attitudes, and barriers associated with telehomecare among caregivers of homebound or bedridden older adults.

The researchers compared the identified codes and discussed the similarities and differences until they reached a consensus on the emergent themes and subthemes. Then, the preliminary results were interpreted with WJ and CA. All authors read and contributed to the manuscript. NVivo (version 12; QSR International) was used for data analysis. Descriptive analysis was used to describe participant characteristics, including frequency, percentage, mean, and SD. The results were presented to caregivers to receive their feedback.

## Results

### Sociodemographic Data of Caregivers

A total of 20 caregivers were recruited, of which 25% (n=5) were male and 75% (n=15) were female, with an average age of 59.5 (SD 14.27) years. Fourteen (70%) of the caregivers were identified as the children of the patients, 15 (75%) caregivers had at least a bachelor’s degree or equivalent level of education, and the average duration of patient care was 3.3 (SD 2.9) years. Table 1 provides a summary of the general information about the caregivers. The average interview duration was 28.45 minutes, ranging from 20 to 47 minutes.

**Table 1:** Sociodemographic data of caregivers (N=20).

Item		Value
**Sex, n (%)**		
	Male	5 (25)
	Female	15 (75)
**Age (years)**		
	Mean (SD)	59.5 (14.27)
	Range	26-87
**Relation to patient, n (%)**		
	Spouse	2 (10)
	Siblings	1 (5)
	Child	14 (70)
	Grandchild	3 (15)
**Highest education, n (%)**		
	Primary school	2 (5)
	Secondary school	3 (15)
	Bachelor’s degree and higher	15 (75)
**Duration of receiving home health care (years)**		
	Mean (SD)	3.3 (2.9)
	Range	1.5-15

### Health Status of Older Adult Patients

The majority (15/20, 75%) of patients were female, with an average age of 86.2 (range 66-95) years. Among these patients, 8 (40%) patients were bedridden and 12 (60%) patients were homebound. The most common comorbidities included hypertension (15/20, 75%), dyslipidemia (7/20, 35%), dementia (7/20, 35%), and stroke (6/20, 30%). Additionally, 45% (n=9) of patients reported needing assistance for using technology, while 40% (n=8) of patients indicated that they were unable to use technology.

### Caregivers’ Expectations, Attitudes, and Barriers Toward Telehomecare

#### Overview

Based on the interviews, it was found that caregivers of older adult patients have specific attitudes and expectations toward taking care of the older adult by using telehomecare. They see it as a valuable service for overall health assessment, although they acknowledge limitations in certain aspects. Communication and device usability challenges were noted, but there is a clear advantage to telehomecare in specific cases compared with in-person visits. Confidence in this service is widespread, yet individual preferences are influenced by family circumstances and situations. Multimedia Appendix 1 shows the framework matrix. The details are provided in the following sections.

#### It is a Service That Provides an Overall Assessment of a Patient’s Health, but Some Aspects May Be Limited

When surveying caregivers’ perceived usefulness toward telehomecare for older adult patients within the comprehensive geriatric assessment framework, differing attitudes emerged. Overall, caregivers displayed a favorable attitude toward telehomecare, despite some limitations in specific areas.

### Physical Assessment

With regard to the physical assessment of the patients, telehomecare can be more limited than in-person home visits. However, caregivers can mitigate this by providing clearer information through photos and videos.

Similar to the case of my mother’s mole which turned into a malignant tumor, it was not noticed clearly through VDO call.

Participant 019

It doesn’t affect me. If grandma has a pain or where there is any wound, the doctor will ask to take pictures or ask her to walk around to observe the symptom through video call.

Participant 014

### Psychological Assessment

Caregivers believe that psychological assessments yield similar outcomes through both telehomecare and in-person visits, as doctors can ask questions and provide privacy for patients during an assessment session.

It’s not different. The doctor asks the same questions, such as “Are you worried about anything?” or “Would you like me to help you with anything?”

Participant 017

### Environmental Assessment

In-person visits offer a broader environmental assessment experience compared with telehomecare, leading to more precise advice on home environment modification. However, caregivers have found ways to use telehomecare effectively, such as walking around with the device.

If the doctor was to visit in person, they would be able to see the home environment and provide advice on how to improve it. If it is stuffy, smelly, unsightly, not suitable for the patient, then the doctor can suggest more than a VDO call. This is because sometimes we don't show the home environment as much.

Participant 013

### Multidisciplinary Care

Telehomecare facilitates engagement with specialists such as dieticians and physiotherapists, thus enhancing patient care.

The telehomecare allows us to have more knowledge from other members of the health care team, especially about how to do physical therapy and how to manage the diet for the patient.

Participant 015

### Goal of Care and Care Planning

Caregivers play a key role in facilitating communication and treatment plan adherence during telehomecare, ensuring patients follow prescribed plans effectively.

We’re able to keep up with the treatment plan even during telehomecare because when the doctor has a video call with my grandma, I always listen and take notes, and then I go over what the doctor has told her. I take notes of it all.

Participant 002

#### Communication and Device and Usability Limitations Are Also Challenges

In telehomecare, despite the content, care processes, and follow-ups remaining the same, challenges still arise. The main barriers are related to communication and issues with the equipment, but most caregivers are capable of handling these challenges.

### Communication

Challenges include low volume from either the patient or health care worker, low patient inattention, and faulty devices, leading to unclear communication. Caregiver presence during sessions can help overcome these barriers.

Grandma’s voice is quite soft. She can’t speak loud making it difficult for the doctor to hear

Participant 002

During telehomecare, if mother is sitting and trying to listen, it may be necessary for a caregiver to be present to help explain things, as she may have a reduced ability to listen and understand due to her age and weakening internal systems.

Participant 015

### Technologies and Signaling Systems

Loss of information may occur in telehomecare, but some caregivers have modified or changed the signaling equipment and environment to enhance it, resolving this issue.

The sound is a bit lacking, but it's probably my Internet. After changing the Wifi, it's better. My mother’s room lacks internet signal.

Participant 007

Barriers? There are some when we use VDO call. Because the phone is old, and we changed the area where we put the phone, so we received a call late. We don’t use Wifi.

Participant 018

### Ability to Use Technology

Older caregivers may encounter difficulties due to their unfamiliarity with technology, affecting their ability to use telehomecare equipment.

I am not good at using the smartphone. Sometimes they ask me to take pictures of this and that area and I don’t really know how to shoot and am not very proficient in using the device.

Participant 013

#### There is an Obvious Advantage of Telehomecare in Limited Cases Rather Than In-Person Home Visits

Despite some challenges and difficulties, telehomecare offers a clear advantage by providing a means of communication in remote areas.

### Scheduling Conflicts

Telehomecare can resolve scheduling conflicts caused by caregiver appointments, enabling remote participation when in-person visits are not possible.

There are times when the older adult patient is not at home during the appointment time. Recently, Grandma was at the center. But we communicated through Line in this group. And then we turn on the camera and talk from 3 different places: the center, the hospital and the office. I can even participate in the call while at work.

Participant 020

### Inconvenient Home Settings

Telehomecare is convenient when the patient’s home is not suitable for visits, for example, the home space is not suitable for the health care team to visit, or traveling to the home of the patient is inconvenient. It is especially effective when assessments rely on the caregiver alone, as outcomes remain similar without the need for travel.

It’s convenient. We don't have to prepare anything. It’s the same. The appointment time doesn’t need to be made, just only when she is unable to sleep.

Participant 017

### Limited Participation of the Patient

It is also effective when assessments rely solely on the caregiver, for example, when the patient has limited participation in conversation, as outcomes remain similar without the need for travel.

...I think it's convenient for both parties. Maybe the patient is taking a nap when the team visit, so eventually the doctor didn't talk to grandma anyway but to me.

Participant 016

#### Everyone is Confident About This Type of Service, but Their Preference Depends on the Family Circumstance and Situation

##### Overview

When asked about transitioning from in-person home visits to telehomecare, all participants (100%) expressed confidence in receiving telehomecare. Caregivers believed it was similar to in-person visits and met their expectations for health care services. For instance, suggestions about how to reduce health risk behaviors, providing physical therapy advice, managing diet, and offering psychological support for both patients and caregivers.

I feel confident because if we have any problems, we can ask just like how we did when the doctor came, but the disadvantage is that the doctor does not see the patient in person but everything else is the same

Participant 016

The preference of individual caregivers toward the in-person home visit versus the telehomecare service model is due to personal experiences and all aspects gained during both forms of services. These can be divided into 3 categories.

##### Preference for In-Person Home Visit

Some caregivers prefer in-person visits because they provide a broader view. These visits allow for a more comprehensive understanding of the patient’s living conditions, environment, evolving symptoms, and overall well-being. In addition, from the direct experience of caregivers, it is easier to identify additional abnormalities during in-person visits that may require treatment. Eight caregivers preferred in-person home visits because they could see more of the overall picture of the patient and the environment.

Because the last time the doctor and the nurse came to visit the house, they gave me notice and later, mother had discovered a mole that protruded into a malignant tumor, we didn't know it. We thought it was a normal wart. Here, in-person home visits are very helpful. because sometimes caregiver doesn't know what it is.

Participant 019

##### Preference of Telehomecare

Two caregivers prefer telehomecare over in-person visits because it is more convenient as they require less preparation. Often, during home visits, the patient may be sleeping and most of the communication is with the caregiver.

She prefers a VDO call because it is convenient for both doctor and patient. Sometimes the patient will be taking a nap. Most of the patients as old as grandma start napping a lot. If the doctor comes and grandma is not talking to him anymore, she’ll take a nap. This is a waste of the doctor’s time.

Participant 016

##### Preference for Both Methods

Five caregivers had similar preferences for both forms of services. They appreciated that both approaches provided an equivalent level of service and treatment, meeting their expectation and goals of health care services during examinations and treatment processes.

I like both. I had no problems with both of them, Through VDO calls, we also get what we need for patient care - medicines and lab results. For me, I don’t have a preference for either method because the specific advantages are different.

Participant 003

Additionally, some of the caregivers had no preference between in-person home visits and telehomecare as they found advantages in both methods depending on the situation, such as the current pandemic situation and the patient’s condition.

It can be both. But now the doctor says that during the COVID pandemic, he still needs to communicate through VDO call as it is good for both sides. As for the in-person home visit, it provides a visual interaction, but during A VDO call, we still have talking, interviewing, asking questions, providing the health care process which is similar kind of work as well.

Participant 014

## Discussion

### Principal Findings

The study discovered that telehomecare is not the same as in-person visits. It may present certain difficulties and barriers, particularly in the aspects of assessment, interaction, and technology use. Despite these barriers, it has the potential to fulfill requirements and bolster trust among those caregivers who provide care to older adults at home to a similar extent as a face-to-face visit. The majority of patients prefer both forms of care, but it is important to adapt use to fit specific circumstances and conditions.

### Comparison With Prior Work

The caregivers of homebound or bedridden older adults expressed positive attitudes toward telehomecare and are prepared to familiarize themselves with this service, comprehending its limitations and the need for its use instead of in-person visits. The previous scope review for patients receiving the telehomecare service at home found that patients desire telehomecare as a supplementary channel, offering easier access to health services from the health care team. This can enhance symptom management and encourage greater self-care [30]. Additionally, 1 study shows that telehomecare patients have greater expectations of telehomecare for the quality and efficiency of patient care, a positive relationship with the team, reduced travel costs [31], and a sense of continuous health monitoring and reassurance from their health care team [32]. If these concerns are addressed in patient care, it will heighten the confidence and satisfaction of both patients and their families.

Moreover, previous studies have found that the majority of participants with a negative attitude toward telehomecare services need more face-to-face health checks than those monitored by telehomecare. This is because they did not have a sense of touch as in in-person visits and were not familiar with using devices. This caused barriers. Conversely, those who are already comfortable with the technology tend to have a more favorable view and see telehomecare as a convenient option, eliminating the need for physically transporting patients to a hospital [8]. These findings align with the results of this study.

### Recommendations for Enhancing Telehomecare Services

Based on these results, there are 4 recommendations for enhancing telehomecare services as provided in the following sections.

#### The Provider Should Enhance the Patients’ or Caregivers’ Skills to Do Self-Health Assessment

Our study results revealed that it is possible to conduct an overall assessment of a patient’s health through telehomecare, but there may be limitations in assessing certain aspects, such as physical assessment. Telehomecare’s limitations make it hard to conduct comprehensive videoconferencing or telephone-based health checks, leading to potential medical errors. To ensure effective patient care, family members and caregivers must be involved [33]. Educating them on symptom recognition and initial assessments can help detect abnormalities early, leading to prompt notification for further assessment by the health care team [23]. It is important to provide training and education to caregivers on how to effectively use telemedicine services, as it can improve their skills in self-observing symptoms and mental health management. Telehomecare can also be used to educate and promote self-care, which can lead to better health outcomes for patients. Various channels, such as phone calls, websites, apps, or chats, can be used to provide additional health care skills to caregivers [34,35].

#### The Health Care Team Should Enhance Their Skills on Telehomecare Services for Patients at Home

A technical problem has been reported as an issue. Therefore, it is crucial for the health care team to possess the necessary skills in telehomecare services to assist patients and their families when they encounter such problems in order to enhance ease of use. It has been suggested that health care teams possess the following skills when delivering telehomecare services [36]: (1) determining when to use telemedicine and assessing the ability of patients and caregivers to use it; (2) proficiency in assessing and caring remotely for patients; (3) effective communication and relationship-building with patients, caregivers, and families; (4) professionalism; (5) basic understanding of information technology; (6) knowledge of laws and privacy protection; (7) ethical considerations; (8) awareness of patient’s safety; and (9) awareness of accessibility and service culture. All of these knowledge and skills required by the health care team impact the provision of effective services which are of utmost importance. In some countries, such as the Netherlands, core competencies have been defined for nurses to ensure the effective implementation of telehealth [37].

The key findings from this study suggest that the health care team should know how to assess the situation and select the appropriate service models, whether it could be in-person home visits or telehomecare based on the experience. Although service users have different preferences, circumstances and necessities should be considered. According to the study by Doraiswamy et al [38], it was noted that telehomecare services had previously been used for noncommunicable diseases. However, during the current pandemic, they have become increasingly important for diagnosis, symptom monitoring, rehabilitation physiotherapy, and reducing the spread of pathogens as well [38]. Despite these advantages once the outbreak situation improves, telehomecare services should only be provided in appropriate cases. Patients with positive attitudes toward telehomecare services; who have used technology and have previously received in-person medical treatment; and have difficulties traveling to the hospital due to distance, finances, or health issues are typically considered suitable for telehomecare services. In addition, the content discussed during the telehomecare services should not be of a sensitive nature, particularly in the case of relationships with the caregiver [39]. It is important to ensure that the patient has a suitable place to receive telehomecare services without distractions and can provide the necessary information to the health care team.

The preferences of patients and their families are a crucial factor in choosing the right telehomecare service. It is important for the physician to consider various aspects, including the patient’s health, family preferences on service models, and the current social situation. Health care providers should engage in conversations with older adult patients and their caregivers regarding the advantages and disadvantages of telehealth, enabling patients to make informed choices between in-person and telemedicine options [23]. This is to make an informed decision and ensure patient satisfaction with the chosen service in the future.

#### The Barriers, Especially Those That Are Fundamental to Providing Telehomecare Services, Should Be Removed as Much as Is Feasibly Possible

In our study, we found that barriers to telehomecare can originate from patients themselves, including soft speaking voices or inadequate communication devices. Technology-related barriers, such as poor signal quality or connectivity issues, may also occur. To mitigate these issues, providers and recipients may need to invest in proper equipment, use a microphone that can absorb speech well, and place equipment in the right position to optimize the internet signal [33]. Additionally, the financial aspect of the service should also be considered, it may be useful to have a system to restore certain devices from the service provider but must be weighed against the expenses of the service provider side as well.

Some caregivers struggle to use communication devices, which can cause interruptions in telehomecare services. Patients may also feel incapable of learning to use these devices, according to a study by Huang et al [8]. Thus, telehomecare teams should be knowledgeable about the devices used, inquire about any concerns, and provide assistance to build confidence in using them. To ensure accessibility to all areas, it is recommended to develop strong internet signal towers to support telehomecare for the national benefit.

Interestingly, this study found that the majority of caregivers for older adult patients were highly educated children, with great potential to use technology better than the patient. However, they may still have limitations in using communication devices and technology [40]. Health care providers should not judge their ability based on age or education, and even frail older adults can be trained with help from nurses and caregivers [41]. Each family should be evaluated on a case-by-case basis, and appropriate assistance provided.

#### Services Should Also Be Provided to Meet Expectations Regarding the Management of Health Problems

Caregivers of older adult patients often have specific expectations from their health care providers, such as recommendations to enhance the patient’s overall health, psychological support, and reducing transportation costs. Our findings support that caregivers perceive telehomecare as useful and prefer it when their expectations are met during the service delivery. Offering services that meet the expectations of patients and their caregivers helps improve the health care aspect and engage in service involvement, which leads to a reduction in hospitalization rates [41,42]. The health care team should strive to meet these expectations to maintain the trust of patients and their families in the telehomecare health care service to the same extent as in an in-person home visit.

### Strengths

The strength of this study is that the study involved caregivers with experience in both in-person home visit and telehomecare, allowing for comparison of both services. The study also focused on in-depth aspects related to caring for the bedridden older adults who may have limitations in receiving services.

### Limitations

However, there are also limitations to this study. For instance, the information obtained through interviews by health care teams or personnel from the hospital may be biased. To mitigate this, the researchers took measures to ensure that the interviewer was not involved in the treatment of any of the patients and caregivers interviewed. The interview process for participants using convenience sampling may involve selecting individuals who are readily available or easy to reach, potentially leading to a sampling bias. However, the data reach its saturation. Additionally, the sex of participants was 75% (n=15) female, which may yield different insights compared with settings with varying sex ratios among caregivers. However, in Thailand, the majority of caregivers for older adults are female, with a prevalence ranging from 70% to 90% [43-45]. This ratio is similar to that in our study. Given the slight tendency for male individuals to hold slightly more favorable attitudes toward technology use than female individuals, although not substantially [46], it is important to note that this sex imbalance among caregivers should not significantly impact the study’s results. The participants of this study were mainly caregivers of older adults with stable chronic illnesses. Caregivers of patients with more complicated illnesses or the terminally ill may have different attitudes or expectations. We did not include the attitudes of the medical service team with regard to this aspect. These attitudes may affect the outcomes of services and treatment as well, which may be subject to further study. Last, conducting a study comparing telehomecare with no care would underscore the significance of home-delivered care. However, since our initial aims did not include a comparison of these 2 populations, we did not collect data from individuals who have not received the service. It may be worthwhile to consider further research involving families of older adults with health conditions that limit their function but are unable to obtain home care services. Nevertheless, obtaining information about this population could be challenging.

### Conclusion

In conclusion, telehomecare is a viable option to supplement in-person home visits. It has the potential to provide quality health care services to patients and allow health care teams to offer treatment and advice remotely. Caregivers have shown a positive attitude toward telehomecare, which is comparable in effectiveness to in-person visits. Despite potential challenges, telehomecare can be a useful alternative in situations where in-person visits are not possible. However, it is important to enhance the skills of caregivers and health care teams to overcome barriers and optimize the use of telehomecare.

## Appendix

Multimedia Appendix 1 Framework matrix.

## Abbreviations

SRQR: Standards for Reporting Qualitative Research

## Data Availability

The data sets generated or analyzed during this study are available from the corresponding author upon reasonable request.

## References

[1] Suriyanrattakorn S, Chang CL (2021). Long-term care (LTC) policy in Thailand on the homebound and bedridden elderly happiness. Health Policy Open.

[2] Ko Y, Noh W (2021). A scoping review of homebound older people: definition, measurement and determinants. Int J Environ Res Public Health.

[3] Bekdemir A, Ilhan N (2019). Predictors of caregiver burden in caregivers of bedridden patients. J Nurs Res.

[4] Schirghuber J, Schrems B (2023). Being wheelchair-bound and being bedridden: two concept analyses. Nurs Open.

[5] Klein S, Hostetter M, McCarthy D (2017). An overview of home-based primary care: learning from the field. Issue Brief (Commonw Fund).

[6] Kim CO, Jang SN (2018). Home-based primary care for homebound older adults: literature review. Ann Geriatr Med Res.

[7] Ergin E, Akin B, Kocoglu-Tanyer D (2022). Effect of home visits by nurses on the physical and psychosocial health of older adults: a systematic review and meta-analysis. Iran J Public Health.

[8] Huang KTL, Lu TJ, Alizadeh F, Mostaghimi A (2016). Homebound patients' perspectives on technology and telemedicine: a qualitative analysis. Home Health Care Serv Q.

[9] Kong DC, Chauhan A, Leung ATA, Chin M Telemedicine in the context of COVID-19- a qualitative study of cancer patients and clinicians. Research Square. Preprint posted online on February 1, 2021.

[10] Radhakrishnan K, Xie B, Jacelon CS (2016). Unsustainable home telehealth: a Texas qualitative study. Gerontologist.

[11] Sethi BA, Sethi A, Ali S, Aamir HS (2020). Impact of coronavirus disease (COVID-19) pandemic on health professionals. Pak J Med Sci.

[12] Khoshrounejad F, Hamednia M, Mehrjerd A, Pichaghsaz S, Jamalirad H, Sargolzaei M, Hoseini B, Aalaei S (2021). Telehealth-based services during the COVID-19 pandemic: a systematic review of features and challenges. Front Public Health.

[13] Pinyopornpanish K, Nantsupawat N, Buawangpong N, Pliannuom S, Vaniyapong T, Jiraporncharoen W (2022). Concerns of home isolating COVID-19 patients while receiving care via telemedicine during the pandemic in the Northern Thailand: a qualitative study on text messaging. Int J Environ Res Public Health.

[14] Finkelstein SM, Speedie S, Hoff M, Demiris G (1999). Tele-homecare: telemedicine in home health care.

[15] Phuthong T, Mangsungnoen N (2017). Factors influencing the elderly intention to use and adopt mobile health services. Veridian E-Journal Silpakorn University.

[16] Wardlow L, Roberts C, Archbald-Pannone L (2023). Perceptions and uses of telehealth in the care of older adults. Telemed J E Health.

[17] Goldberg EM, Lin MP, Burke LG, Jiménez FN, Davoodi NM, Merchant RC (2022). Perspectives on telehealth for older adults during the COVID-19 pandemic using the quadruple aim: interviews with 48 physicians. BMC Geriatr.

[18] Bowles KH, Baugh AC (2007). Applying research evidence to optimize telehomecare. J Cardiovasc Nurs.

[19] Mangin D, Parascandalo J, Khudoyarova O, Agarwal G, Bismah V, Orr S (2019). Multimorbidity, eHealth and implications for equity: a cross-sectional survey of patient perspectives on eHealth. BMJ Open.

[20] Shin HR, Um SR, Yoon HJ, Choi EY, Shin WC, Lee HY, Kim YS (2023). Comprehensive senior technology acceptance model of daily living assistive technology for older adults with frailty: cross-sectional study. J Med Internet Res.

[21] Ma Q, Chan AHS, Teh PL (2021). Insights into older adults‘ technology acceptance through meta-analysis. Int J Hum Comput Interact.

[22] Cook EJ, Randhawa G, Guppy A, Sharp C, Barton G, Bateman A, Crawford-White J (2018). Exploring factors that impact the decision to use assistive telecare: perspectives of family care-givers of older people in the United Kingdom. Ageing Soc.

[23] Raj M, Iott B, Anthony D, Platt J (2022). Family caregivers' experiences with telehealth during COVID-19: insights from Michigan. Ann Fam Med.

[24] O'Brien BC, Harris IB, Beckman TJ, Reed DA, Cook DA (2014). Standards for reporting qualitative research: a synthesis of recommendations. Acad Med.

[25] Vasileiou K, Barnett J, Thorpe S, Young T (2018). Characterising and justifying sample size sufficiency in interview-based studies: systematic analysis of qualitative health research over a 15-year period. BMC Med Res Methodol.

[26] Gale NK, Heath G, Cameron E, Rashid S, Redwood S (2013). Using the framework method for the analysis of qualitative data in multi-disciplinary health research. BMC Med Res Methodol.

[27] Marangunić N, Granić A (2015). Technology acceptance model: a literature review from 1986 to 2013. Univ Access Inf Soc.

[28] Chadborn NH, Goodman C, Zubair M, Sousa L, Gladman JRF, Dening T, Gordon AL (2019). Role of comprehensive geriatric assessment in healthcare of older people in UK care homes: realist review. BMJ Open.

[29] Stoop A, Lette M, van Gils PF, Nijpels G, Baan CA, de Bruin SR (2019). Comprehensive geriatric assessments in integrated care programs for older people living at home: a scoping review. Health Soc Care Community.

[30] Steindal SA, Nes AAG, Godskesen TE, Dihle A, Lind S, Winger A, Klarare A (2020). Patients' experiences of telehealth in palliative home care: scoping review. J Med Internet Res.

[31] Chang JY, Chen LK, Chang CC (2009). Perspectives and expectations for telemedicine opportunities from families of nursing home residents and caregivers in nursing homes. Int J Med Inform.

[32] Husebø AML, Storm M (2014). Virtual visits in home health care for older adults. ScientificWorldJournal.

[33] Fageir S, Osman O, Addison C (2023). A closer look at dementia patients’ barriers to telemedicine utilization during the COVID-19 pandemic. Eur J Env Public Hlt.

[34] Graven LJ, Glueckauf RL, Regal RA, Merbitz NK, Lustria MLA, James BA (2021). Telehealth interventions for family caregivers of persons with chronic health conditions: a systematic review of randomized controlled trials. Int J Telemed Appl.

[35] Kruse C, Betancourt J, Ortiz S, Luna SMV, Bamrah IK, Segovia N (2019). Barriers to the use of mobile health in improving health outcomes in developing countries: systematic review. J Med Internet Res.

[36] Galpin K, Sikka N, King SL, Horvath KA, Shipman SA, AAMC Telehealth Advisory Committee (2021). Expert consensus: telehealth skills for health care professionals. Telemed J E Health.

[37] van Houwelingen CTM, Moerman AH, Ettema RGA, Kort HSM, Cate OT (2016). Competencies required for nursing telehealth activities: a Delphi-study. Nurse Educ Today.

[38] Doraiswamy S, Abraham A, Mamtani R, Cheema S (2020). Use of telehealth during the COVID-19 pandemic: scoping review. J Med Internet Res.

[39] Sturesson L, Groth K (2018). Clinicians' selection criteria for video visits in outpatient care: qualitative study. J Med Internet Res.

[40] Rojanasumapong A, Jiraporncharoen W, Nantsupawat N, Gilder ME, Angkurawaranon C, Pinyopornpanish K (2021). Internet use, electronic health literacy, and hypertension control among the elderly at an urban primary care center in Thailand: a cross-sectional study. Int J Environ Res Public Health.

[41] Finkelstein SM, Speedie SM, Zhou X, Potthoff S, Ratner ER (2011). Perception, satisfaction and utilization of the VALUE home telehealth service. J Telemed Telecare.

[42] Bowles KH, Hanlon AL, Glick HA, Naylor MD, O'Connor M, Riegel B, Shih NW, Weiner MG (2011). Clinical effectiveness, access to, and satisfaction with care using a telehomecare substitution intervention: a randomized controlled trial. Int J Telemed Appl.

[43] Pinyopornpanish K, Wajatieng W, Niruttisai N, Buawangpong N, Nantsupawat N, Angkurawaranon C, Jiraporncharoen W (2022). Violence against caregivers of older adults with chronic diseases is associated with caregiver burden and depression: a cross-sectional study. BMC Geriatr.

[44] Pinyopornpanish K, Soontornpun A, Wongpakaran T, Wongpakaran N, Tanprawate S, Pinyopornpanish K, Nadsasarn A, Pinyopornpanish M (2022). Impact of behavioral and psychological symptoms of Alzheimer's disease on caregiver outcomes. Sci Rep.

[45] Sanprakhon P, Chaimongkol N, Hengudomsub P (2022). Relationships between caregiving stress and sleep quality among family caregivers of older adults with dementia in Thailand. Belitung Nurs J.

[46] Cai Z, Fan X, Du J (2017). Gender and attitudes toward technology use: a meta-analysis. Comput Educ.

