# Supplementary material for: Expectation, Attitude, and Barriers to Receiving Telehomecare Among Caregivers of Homebound or Bedridden Older Adults: Qualitative Study
Source: JMIR Aging. 2024 Feb 7;7:e48132. doi: 10.2196/48132 (PMC10882467; doi:10.2196/48132)
Supplement: Multimedia Appendix 1 [file aging_v7i1e48132_app1.docx]

**Multimedia Appendix 1.** Framework Matrix.

| **Themes** | **Subthemes** | **Categories** | **Quote** |
| --- | --- | --- | --- |
| It is a service that provides an overall assessment of a patient's health, but some aspects may be limited. (Perceived usefulness/expectation) | Physical Assessment | More limited with telehomecare | “Video calls, they can't see, so they can't examine. They can only ask about symptoms. It can make a slight difference... In video calls, they can't physically touch the patient.” (004)  “In some cases, because when they come to visit, they will have equipment to measure oxygen levels and check the wound, like this. So, in situations like this, we can only show things through the camera like this.” (007)  "Similar to the case of my mother's mole which turned into a malignant tumor, it was not noticed clearly through VDO call.” (019) |
|  |  | Possibility to provide clearer information through photos and videos. | “Regarding examining the patient's body, I have to start from head to toe, approximately like that. But if the doctor comes, they can see the whole picture and provide specific observations like, "Uncle, you've lost weight," "You have gained weight," "There's a bone issue here and there," and so on. It might require some coordination, like if there's something specific to check, can we use video at that spot? This way, we can get a clearer view and address issues more effectively.” (006)  “It doesn't affect me. If grandma has a pain or where there is any wound, the doctor will ask to take pictures or ask her to walk around to observe the symptom through video call.” (014)  “For remote physical examinations done over the phone, communication is facilitated through the caregiver. Grandma describes her symptoms, and the doctor might request to see her feet, joints, and other areas to check for signs of stiffness or issues. In such cases, we can use the camera to inspect these things and send the images to the doctor.” (020) |
|  | Psychological Assessment | Similar assessment regarding psychological issues | “It's not different. The doctor asks the same questions, such as "Are you worried about anything?" or "Would you like me to help you with anything?" (017)  “Similar assessment for psychological status was done.” (007, 011, 014) |
|  |  | Possibility to keep privacy | “The doctor will separately ask us similar questions, whether they come for an in-person home visit or conduct the examination online. That's because when they visit at home, there are usually my siblings or other family members present. The doctor will ask me privately. It's the same with phone calls; once the conversation is done for all, the doctor will proceed to talk to others separately to maintain privacy. It works similarly in both cases.” (020) |
|  | Environmental Assessment | Broader environmental assessment with in-person visits | “In a wider view, the doctor won't be able to see the general surroundings because the frame of the view is limited to the camera we're looking through. However, in the broader environment, the doctor may not see it.” (006)  “If the doctor was to visit in person, they would be able to see the home environment and provide advice on how to improve it. If it is stuffy, smelly, unsightly, not suitable for the patient, then the doctor can suggest more than a VDO call. This is because sometimes we don't show the home environment as much.” (013)  “But with a VDO call, it doesn't capture everything. It only shows the inside... Like in person, the doctor can walk around and examine more extensively. It's easier to explain things during a conversation. But with a video call, it's limited to certain points, and the perspective is narrower.” (014) |
|  |  | No different for bed ridden patient | “It's not different, really. In grandma's condition, she stays in bed, and there haven't been any changes. She can't walk or do much on her own. She can manage her daily routines to some extent but mostly stays in bed. The environment is pretty much the same.” (010) |
|  |  | Doctor can request | “It depends on the doctor's request. During our video calls, we only capture the area around the bed. It shows the patient's face, skin, and other basic things like checking for any issues with blood circulation or slowness, but it doesn't provide a view of the surroundings.” (007)  “The doctor can request to see the environment, the bathroom, and such things, to check if there are any devices for your grandma to use to help herself.” (014) |
|  | Multidisciplinary care | Telehomecare facilitates engagement with specialists | “The telehomecare allows us to have more knowledge from other members of the health care team, especially about how to do physical therapy and how to manage the diet for the patient. (015)” |
|  | Goal of care and care planning | Caregivers as a facilitator of the communication and following the plan | "We're able to keep up with the treatment plan even during telehomecare because when the doctor has a video call with my grandma, I always listen and take notes, and then I go over what the doctor has told her. I take notes of it all." (002)  “If the caregivers provide all the information, there won't be much difference. But if the caregivers don't observe closely, or if they don't check regularly, they might not know what issues the patient faces each day. For instance, I ask the caregivers every day about how your father is doing, how he sleeps, what he eats, if there are any urinary retention or blood-related issues. Once we have enough information, we can consult with the doctor.” (006) |
|  |  | No difference if well advanced planning or stable condition | “There isn't much of a difference in planning for now because the condition of the patient hasn't worsened yet. If there are any problems, we can always call for assistance.” (007)  “We've planned with the doctor all along. If grandma has any discomfort or something like that, we'll take her to the hospital. It'll make her most comfortable. We've already prepared a plan, so I think video calls don't differ much from the treatment plan.” (010) |
| Communication and device and usability limitations are also challenges (Perceived ease of use/ Barrier) | Communication | Low volume leads to unclear communication | “Grandma’s voice is quite soft. She can’t speak loud making it difficult for the doctor to hear” (002)  “Patients with poor hearing is unable to respond by phone. If you visit the home in-person, speak loudly so the patient can interact with the doctor and you should be able to express your feelings better.” (008)  “During telehomecare, if mother is sitting and trying to listen, it may be necessary for a caregiver to be present to help explain things, as she may have a reduced ability to listen and understand due to her age and weakening internal systems.” (015) |
|  |  | Convenience to talk when in-person | “When the doctor comes (in-person visit), he (the patient) will be able to talk and discuss easily, right?” (001)  “if the doctor comes in-person, he/she will be a little more friendly.” (004)  “With in-person visit, grandma can discuss better with the doctor.  With video calls, it's just sitting and talking to the screen, and the level of attention might be lower compared to an actual visit. In addition, when we talk, we have to take turns looking at the screen. It's not a broad view.” (014) |
|  | Technologies and signaling systems | Problem regarding technology or communication signaling (Can be solved by modifying or changing the signaling equipment and environment) | “My phone had a problem at that time; it wasn't working. We had to take it to the shop to see why it wasn't working, and they fixed the system for us.” (005)  “The sound is a bit lacking, but it's probably my Internet. After changing the Wifi, it's better. My mother's room lacks internet signal." (007)  “My phone is a landline type. It just rings loudly, and if it's a mobile phone, we might not hear it, so it's more convenient with my phone.” (009)  “Barriers? There are some when we use VDO call. Because the phone is old, and we changed the area where we put the phone, so we received a call late. We don’t use Wifi.” (018) |
|  | Ability to use technology | Encounter difficulties by caregivers | “The problem is that if some patients don't have children or caregivers who are tech-savvy enough to support them in this aspect, it might be a bit challenging. It's not just about having a phone; if they can't use video calls, it can become a problem. But if the children or caregivers have the technology to support this system, it's good.” (006)  “I am not good at using the smartphone. Sometimes they ask me to take pictures of this and that area and I don’t really know how to shoot and am not very proficient in using the device… Maybe I need to upgrade myself. I need to learn how to use the phone better because it's not really my thing.” (013) |
| There is an obvious advantage of telehomecare in limited cases rather than in-person home visits. (Perceived usefulness) | Scheduling conflicts | Enabling remote participation | “We can call and talk without wasting the doctor's time. Sometimes I feel sorry for the doctor because sometimes I'm not at home, and when they come, they have to look for me because I like going outside. Once we switched to the phone, my mother can surely receive the call.” (018)  “There are times when the older adult patient is not at home during the appointment time. Recently, Grandma was at the center. But we communicated through Line in this group. And then we turn on the camera and talk from 3 different places: the center, the hospital and the office. I can even participate in the call while at work.” (020) |
|  | Inconvenient home settings | Less preparation | “It's convenient. We don't have to prepare anything. It's the same. The appointment time doesn’t need to be made, just only when she is unable to sleep.” (017)  “If they inform (that they will visit) in advance, I can do some cleaning and checking beforehand, making it more presentable. But it might not be 100% clean because my eyesight is not good.” (009) |
|  | Limited participation of the patient | Assessments rely solely on the caregiver | “...I think it's convenient for both parties. Maybe the patient is taking a nap when the team visit, so eventually the doctor didn't talk to grandma anyway but to me.” (016) |
| Everyone is confident about this type of service, but their preference depends on the family circumstance and situation. (Perceived ease of use/ Attitude) | Preference of in-person home visit | Provide a broader view and identify additional abnormalities | “I prefer visits in person more because I think the doctor can see more things.” (001)  “I prefer real home visits much more because the doctor really takes good care. When they come, they really see how my mother is doing, the actual condition, and the environment as well. You get to see a lot more than with a video call, from a broader perspective.” (013)  “Because the last time the doctor and the nurse came to visit the house, they gave me notice and later, mother had discovered a mole that protruded into a malignant tumor, we didn't know it. We thought it was a normal wart. Here, in-person home visits are very helpful. because sometimes caregiver doesn't know what it is.” (019) |
|  |  | Better concentration by caregivers | “I prefer it when the doctor visits our home more because in one way, I get to know the details better. From my mother's perspective, she feels better too, and I'm reassured that it's been a while since she visited the doctor. Video calls sometimes have gaps, and sometimes when I'm not concentrate well, I might miss some details. I might forget things." (015) |
|  | Preference of telehomecare | Convenience for both doctor and patient | “She prefers a VDO call because it is convenient for both doctor and patient. Sometimes the patient will be taking a nap. Most of the patients as old as grandma start napping a lot. If the doctor comes and grandma is not talking to him anymore, she'll take a nap. This is a waste of the doctor’s time." (016)  “I like video calls because there's no need to prepare anything, and it's just like normal.” (017) |
|  | Preference for both methods | Equivalent level of service and treatment | “I like both. I had no problems with both of them, Through VDO calls, we also get what we need for patient care - medicines and lab results. For me, I don't have a preference for either method because the specific advantages are different.” (003)  “Both options are fine because whether you visit in person or communicate via phone call, it's equally good.” (018) |
|  | No preference | Advantages in both methods depending on the situation or patient condition | “It's convenient in this (pandemic) situation.” (001, 004)  “It's a good idea to visit patients who don't have any serious or severe illnesses. If it's something with severe or worsening symptoms, it's best for the patient to meet with a doctor in person.” (002)  “It can be both. But now the doctor says that during the COVID pandemic, he still needs to communicate through VDO call as it is good for both sides. As for the in-person home visit, it provides a visual interaction, but during A VDO call, we still have talking, interviewing, asking questions, providing the healthcare process which is similar kind of work as well.” (014)  “In some cases, like when someone is at a care facility, in-person visiting at home care facility may not be convenient (or not possible). In such situations, a phone call or video call could be an option for communication. However, if an in-person visit is necessary, taking the patient back home before the appointment is also an option.” (020) |
